# Brain activity and cognition: a connection from thermodynamics and information theory

**DOI:** 10.3389/fpsyg.2015.00818

**Published:** 2015-06-16

**Authors:** Guillem Collell, Jordi Fauquet

**Affiliations:** ^1^Sloan School of Management, Massachusetts Institute of TechnologyCambridge, MA, USA; ^2^Department of Psychobiology and Methodology of Health Sciences, Universitat Autònoma de BarcelonaBarcelona, Spain; ^3^Research Group in Neuroimage, Neurosciences Research Program, Hospital del Mar Medical Research Institute, Barcelona Biomedical Research ParkBarcelona, Spain

**Keywords:** free energy, negentropy, brain thermodynamics, cognitive models, information theory

## Abstract

The connection between brain and mind is an important scientific and philosophical question that we are still far from completely understanding. A crucial point to our work is noticing that thermodynamics provides a convenient framework to model brain activity, whereas cognition can be modeled in information-theoretical terms. In fact, several models have been proposed so far from both approaches. A second critical remark is the existence of deep theoretical connections between thermodynamics and information theory. In fact, some well-known authors claim that the laws of thermodynamics are nothing but principles in information theory. Unlike in physics or chemistry, a formalization of the relationship between information and energy is currently lacking in neuroscience. In this paper we propose a framework to connect physical brain and cognitive models by means of the theoretical connections between information theory and thermodynamics. Ultimately, this article aims at providing further insight on the formal relationship between cognition and neural activity.

## Introduction

The brain is a thermodynamic device aimed at processing information. Consequently, brain activity has often been modeled in thermodynamic terms (La Cerra, 2003; Varpula et al., 2013) and cognitive processes in information terms (Anderson, 1996; Friston, 2010). These two different approaches, separately, yield accurate descriptions of brain and cognitive processes. However, their unification would greatly increase our understanding of how brain activity is related to cognition and in turn would benefit both perspectives (Collell and Fauquet, 2014). Critically, there are deep theoretical connections between information theory and thermodynamics. Some of the classical links between the two disciplines are the Landauer limit (Landauer, 1961), the energetic cost of processing information (Bennett, 1982), the Gibbs and Boltzmann formulas and the concepts of entropy and negentropy (Schrodinger, 1944; Brillouin, 1953). Interestingly, entropy is a central concept in both information theory and thermodynamics. Even though it is a measure of a different quantity in each theory, these quantities exhibit important theoretical relationships, as will be discussed below. In fact, several authors suggested that the relationship between thermodynamics and information theory is even stronger and claim that the laws of thermodynamics are nothing but theorems in information theory (Rothstein, 1951; Brillouin, 1953). Notably, the aforementioned connections can serve as a set of powerful tools to unify both, thermodynamic-based brain models and information-based models of cognition (Collell and Fauquet, 2014). For instance, it has yet to be studied whether the equations of both modelizations are consistent. In this sense, if a thermodynamic-based model predicts a change of the system in a certain direction by considering energetic measures, an information-based model should predict a change in the same direction if these thermodynamic measures were translated into information.

In order to briefly introduce the relationship between information and physics, let us suggest the following thought experiment proposed in 1867 which is known as Maxwell's demon paradox. In our opinion, this paradox is not only useful to understand the relationship between thermodynamics and information theory, but also to gain insight on how mind computations are linked to energy and information. In short, consider a container divided in two parts, A and B, both filled with the same gas at equal temperatures. A tiny demon is guarding a trapdoor between A and B and when a faster-than-average molecule is moving toward A, he allows it to pass by briefly opening the trap and closing it immediately. Similarly, he allows slower-than-average molecules to pass to B. After a while doing this task, the temperature, i.e., average molecular speed, will be higher in A than in B. This implies that the entropy (disorder, for now) of the system would have decreased, leading to an apparent violation of the second law of thermodynamics, which states that entropy can only increase for an isolated system. Afterwards, this temperature difference can be used to produce work (Feynman et al., 1998). Maxwell's demon paradox was not considered completely solved until more than a century after its proposal. This solution will be disclosed and briefly commented on afterwards. Remarkably, even without ever having seen any formalization of the concepts of information and physical entropy before, one can intuitively realize from this thought experiment that “knowledge” about the system (cold vs. hot molecule) can somehow be translated into useful energy. The converse, namely the fact that energy can be transformed into information, was perhaps clear and widely accepted from the beginning since in our digital age it does not come as a surprise for anyone that computers consume energy in order to encode or erase data.

**Figure 1:**
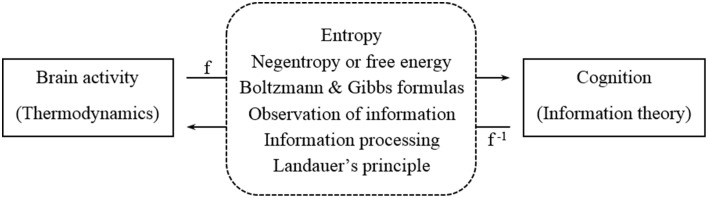
**Connection between brain activity (left) and cognitive models (right) by means of the classical links between thermodynamics and information theory (middle)**.

Several brain and cognitive models have been proposed so far from the thermodynamic viewpoint (Kirkaldy, 1965; La Cerra, 2003; Varpula et al., 2013) as well as from the information theory framework (Anderson, 1996; Friston and Stephan, 2007). However, a connection between information theory and thermodynamics has not been formalized in neuroscience yet (Del Castillo and Vera-Cruz, 2011; Collell and Fauquet, 2014). This article aims at further elaborating the idea that we introduced in a previous paper (Collell and Fauquet, 2014), namely to develop a suitable framework to connect information-based models of cognition and thermodynamic models of the brain. In this respect, the main contribution of this work is studying this relationship from a new perspective, that is, from the classical connections between information theory and thermodynamics (see Figure 1). Ultimately, this paper aims at expanding the formal connection between physical brain activity and cognition.

This paper is structured as follows. In the following section, we analyze how thermodynamics is linked to brain activity and we present some relevant physically-principled models. Next, we present Friston's information-based model and then we propose a list of different means to obtain information measures from brain and cognitive activity. In the next section, we discuss the classical connections between information theory and thermodynamics and afterwards we analyze how these connections should be applied within the brain context. Then, we establish theoretical bridges between the previously described brain and cognitive models by applying the classical links between information theory and thermodynamics. Next, we suggest how the question of spontaneity—applied to brain and cognitive activity—can be addressed from the framework proposed here. Finally, in the discussion, we summarize and analyze the contributions of our approach and we propose future lines of research.

## Thermodynamics and brain activity

### Overview of thermodynamics of open dissipative systems

A core concept in thermodynamics is *entropy*. It is often regarded as the degree of disorder of the system, but it equals to the amount of energy dissipated in form of molecular vibration that cannot be used to produce work, according to its precise definition (Feynman et al., 1965). For example, recalling Maxwell's demon context, there is the same amount of energy in the initial as in the final situation since there is the same total amount of molecular vibration, but in the first scenario it was not possible to use this energy to produce work. The first formal definition of physical entropy (*S*, henceforth) was proposed by Classius in 1856. The entropy change Δ*S* between two states a_1_ and a_2_ corresponds to

$$△S=\int_{a_{2}}^{a_{1}}\frac{\delta Q}{T}$$

where δ*Q* stands for the heat increase and *T* is the system's absolute temperature. Physical entropy is expressed in Joules/Kelvin (J/K) in international units (Feynman et al., 1965).

As briefly outlined above, the second *law of thermodynamics* states that the entropy of any isolated system can only increase, except for small random fluctuations according to its probabilistic formulation. More formally, this principle can be expressed as *dS*/*dt* ≥ 0 (Prigogine, 1978). In this respect, a system is called isolated if it does not exchange neither matter nor energy with the environment and is termed open if it exchanges both. Remarkably, the second law of thermodynamics completely characterizes *spontaneous processes*, i.e., those processes that occur without external help. Thus, these can only occur if the overall result is an increase of entropy in the universe. Therefore, unlike energy, which can be neither created nor destroyed (first law of thermodynamics), entropy can only be created but not destroyed (second law) in an isolated system.

At this point, a fundamental question naturally arises in order to understand the thermodynamics of life, and in particular of the brain: how is it possible that living systems tend to evolve toward states of lower entropy, i.e., higher organization? This is, as it should be, not a real but just an apparent contradiction with the second law of thermodynamics. First, let us recall that the second law is formulated for isolated systems and, by contrast, living systems are the canonical example of open systems. Hence, in order to decrease the entropy of any living system it must be increased elsewhere, giving still as a result an overall increase in the universe. For example, there is an increase of order during the DNA synthesis, but at the expense of breaking adenosine triphosphate (ATP) molecules (i.e., increasing disorder), which still entails an overall increase of entropy. Critically, a fundamental law of living systems states that if entropy reaches a certain threshold the structure and functionality of the organism will be endangered (Schrodinger, 1944). Thus, any important increase of entropy within the system must be promptly eliminated through its boundaries. For example, cellular respiration not only produces ATP molecules, i.e., useful chemical energy, but also a waste product that must be expelled from the cell afterwards.

The concept of *negentropy* or *free energy* stems naturally from the concept of entropy as its “opposite” idea. It was introduced by Schrodinger (1944) to provide a suitable framework to study the thermodynamics of living systems. He initially called it negative entropy since, in the physical sense, it can be interpreted as the amount of energy available to produce work, as opposed to entropy. For example, there is negentropy stored in a neuron before spiking (mainly ATP) or when there is a temperature gradient, as in Maxwell's demon context. Physical negentropy can be described by the Helmholtz free energy *F_H_* formula (Prigogine, 1978):

(1) $$F_{H}=U-TS$$

where *U* is the total internal energy of the system and *T* its temperature.

### Thermodynamic models of the brain

In general, thermodynamic models of brain activity exhibit two important common features: (1) the brain is regarded as a device that eliminates entropy through its boundaries and is supplied by a source of free energy, mainly chemical in form of ATP; and, (2) the second law of thermodynamics is considered as the main principle that drives brain activity (Kirkaldy, 1965; La Cerra, 2003; Del Castillo and Vera-Cruz, 2011; Varpula et al., 2013).

**Figure 2:**
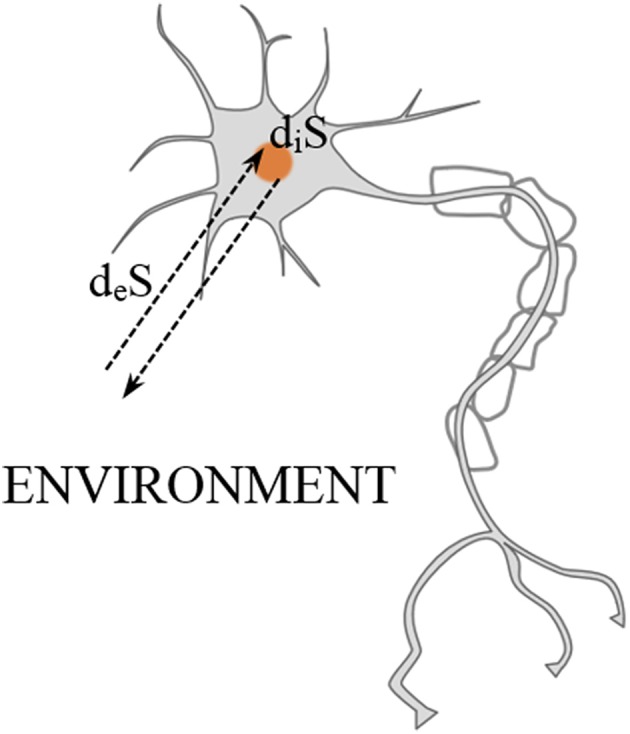
**Entropic exchange between the outside and the inside of a neuron. Adapted from Prigogine (1978)**.

Considering the entropic constraints that living systems must fulfill in order to preserve life, it is convenient to apply the following approach proposed by Prigogine (1978). That is, splitting the total entropy variation *dS* into an increase of entropy inside *d_i_S* and an entropy exchange *d_e_S* through the boundaries of the system, e.g., a brain region or a neuron (see Figure 2).

Importantly, since the second law of thermodynamics must be satisfied inside of the system, *d_i_S* > *0* must be immediately obtained after every neural process. However, this entropy gain must be eliminated afterwards through the neuron's boundaries. This can be formulated (Del Castillo and Vera-Cruz, 2011) as

$$\frac{d_{i}S}{dt}<-\frac{d_{e}S}{dt}.$$

It is a fundamental property of self-organizing systems that when there is an increase of entropy beyond the limit that the current system's structure can dissipate, new structures, such as a cognitive reorganization, may emerge (Prigogine, 1978; Stephen et al., 2009). In addition, in order to recover the capacity to produce work, e.g., to transmit a new train of neural spikes, there must be an inflow of free energy (ATP) into the neuron or brain region. This free energy is provided by a blood flow transporting oxygen and glucose that will be used to restore the ATP quantities (Howarth et al., 2010). This is captured by the following equation which describes the local entropic exchange rate for open dissipative systems in general, and, in particular, for the brain (Del Castillo and Vera-Cruz, 2011)

(2) $$\frac{dS}{dt}=\frac{d_{i}S}{dt}+\frac{d_{e}S}{dt}$$

For a more detailed description, it may be convenient to break the *d_i_S/dt* term into the different irreversible processes and forces involved, that is

(3) $$\frac{d_{i}S}{dt}=\sum_{\rho }J_{\rho }X_{\rho }$$

where *J*_ρ_ are the rates of various ρ irreversible processes (chemical reactions, heat flow, diffusion, etc…) and their corresponding generalized forces *X*_ρ_ (affinities, gradients of temperature, gradients of chemical potential, etc…).

It is important to notice that the above description holds for both, systems in equilibrium and for those in which the local equilibrium hypothesis is satisfied. The hypothesis states that, at a given instant, a system out of equilibrium can be treated as a homogeneous system in equilibrium—where a system is termed homogeneous if its properties do not depend on the position. In other words, the local equilibrium assumes a system split in a number of cells which are sufficiently large for microscopic fluctuations to be negligible but at the same time sufficiently small for equilibrium to be a good approximation in each individual cell. Thus, at a given instant of time, equilibrium is achieved in each individual cell (Lebon et al., 2008). The local equilibrium hypothesis holds for those processes in which the microscopic timescale is substantially shorter than the macroscopic timescale. For example, a microscopic time corresponding to the interval between two successive particle collisions, say 10^−12^ s, compared to a macroscopic process that lasts for, say about 1 s. However, in a number of far from equilibrium processes the local equilibrium hypothesis is not satisfied. This occurs when the microscopic and macroscopic timescales are comparable. For example, in processes involving slow relaxation times such as polymer solutions or superconductors; or in high frequency phenomena such as ultrasound propagation in dilute gases or neutron scattering in liquids. However, if the system is coupled to other variables that relax faster, then thermodynamic quantities such as temperature or entropy can still be defined in situations far beyond equilibrium (Vilar and Rubí, 2001). Thus, the system can still be described. The basic idea is to increase the dimensionality by including other degrees of freedom with smaller relaxation times. For example, in a diffusion process, Vilar and Rubí (2001) proposed to add velocity as a new variable, although this need not always be the case. In conclusion, for an accurate description of any neural processes involving variables with either slow relaxation times or high-frequencies, the extended irreversible thermodynamics setting needs to be considered.

Varpula et al. (2013) modeled neural electrical activity with a version of the second law of thermodynamics as a central assumption. The model states that consumption of free energy in the least possible time is the main principle driving electrical brain activity. In other words, electrical flows within the neural network will search themselves for the pathways that consume more free energy in the least time. In this sense, the neural network is considered the register of remembrance that can be retrieved, consolidated or reorganized by activating certain paths, analogous to a river that erodes its landscape and affects its own flow. Thus, memories are potentiated by flushing specific energetic flow patterns again and again, and will fade if these paths are not reinforced. Notably, this model is capable of explaining a great number of brain processes such as learning, association or memory retrieval by just taking into account the second law of thermodynamics.

It is worth pointing out that several neuroimaging techniques can be used to measure the energetic activity in the brain. A widely used method is functional magnetic resonance imaging (fMRI). It is based on obtaining measures of the blood oxygen level dependent (BOLD) signal, which is an indicator of changes in the blood flow. These metabolic changes indirectly reflect the underlying neural activity. Another well-known method to obtain such measures is electroencephalography (EEG). In particular, with this method recordings of neural electrical activity are obtained by measuring differences in voltage potentials from multiple electrodes distributed along the scalp. Finally, single cell recordings is the most accurate choice to measure the energetic activity of a single neuron. This method is based on measuring the action potentials of a single neuron using a microelectrode inserted in the brain. However, it is an invasive method that cannot be applied in human research. Thus, the best choice will greatly depend on the situation and object of study.

## Information theory and cognition

Before presenting Friston's information-based model we will briefly outline some important definitions and results from information theory. Shannon's information theory was firstly formulated in 1948 in the context of communication, where the scenario was composed of a sender, a receiver and a message sent through a channel (Shannon, 1948). The information of a symbol *x_i_* is defined as

$$I\left(x_{i}\right)=-log_{2}p(x_{i})$$

where *p(x_i_)* corresponds to the probability of occurrence of the symbol *x_i_* in a given message. When the base of the logarithm above is 2, the information is expressed in bits. Thus, *I(x_i_)* is the necessary amount of bits to encode a message composed of just the symbol *x_i_*. The more unlikely *x_i_* is, the more bits will be needed. Alternatively, *I(x_i_)* can be understood as the amount of “surprise” of observing *x_i_*_._

Shannon entropy is represented by *H* and corresponds to the expected information or uncertainty per symbol in a message composed of *N* different symbols *X* = {*x_i_*}, *i* ∈ *{1, …, N}* each of them appearing with probability *p*(*x_i_*). That is,

(4) $$H\left(X\right)=-\sum_{i}p\left(x_{i}\right)log_{2}p(x_{i}).$$

In the context of a noiseless channel, a fundamental result in information theory is the so-called Shannon's source coding theorem. It states that a message cannot be compressed below its entropy bound, without losing information.

### Friston's information-based model

In our opinion, the recent contribution of Friston's information-based model (Friston and Stephan, 2007; Friston, 2010) is especially noteworthy. It offers a description of a wide range of cognitive processes such as perception, learning or inference and is particularly accurate in explaining perceptual processes (Moran et al., 2013).

The core concept of Friston's theory is termed *free energy*. In this case, free energy F is defined as the upper bound of “surprise” or log-evidence –*ln p*($\tilde{s}$|*m*) associated with receiving a sensory input $\tilde{s}$ and having a model of the world m. Free energy is nothing but surprise plus a positive term, the so-called Kullback-Liebler divergence or cross entropy; that is,

(5) $$F=-\ln p\left(\tilde{s}|m\right)+D(q(\vartheta |\mu )||p(\vartheta |\tilde{s}))$$

where the ϑ is an unknown quantity that caused the sensory state $\tilde{s}$, and μ stands for the internal states of the brain. The individual is assumed to encode a probabilistic representation of the world in the internal states μ. The term *D*(*q*(ϑ|μ)||*p*(ϑ|$\tilde{\text{s}}$)) is the cross entropy, that is, the divergence between the recognition density *q*(ϑ|μ) and the true distribution *p*(ϑ|$\tilde{\text{s}}$) of the causes ϑ. The recognition density corresponds to the agent's probabilistic representation of the causes of the input $\tilde{s}$. Conceptually, free energy (Equation 5) can be regarded as: *F* = *Surprise* + *Cross Entropy*.

The central principle of Friston's theory is the so-called *free energy minimization*. That is, in order to preserve life, self-organizing biological agents should avoid surprising states. For this purpose, their free energy must be minimized. For example, a fish would minimize its free energy by avoiding the surprising state of being out of water (Friston, 2010). Critically, the agent can minimize the cross entropy term by changing its internal representations, i.e., by changing the recognition density into a better approximation of the true distribution. Through active inference, i.e., optimizing perception, the agent updates its internal representations by Bayesian inference, leading to a subsequent minimization of the prediction error. In this sense, the system implicitly encodes a probabilistic model of the environment. Critically, active inference (i.e., changing expectations) and action (changing its configuration and avoiding surprising encounters) are the two possible mechanisms by which an organism can minimize its free energy.

Interestingly, through algebraic manipulation of Equation (5), the following reformulation of free energy can be obtained:

(6) $$F=-{\langle \ln p\left(\tilde{s},\vartheta |m\right)\rangle }_{q}+{\langle \ln q(\vartheta |\mu )\rangle }_{q}$$

where the term −<ln*p*($\tilde{\text{s}}$, ϑ|m) >_*q*_ is the expected surprise [under the density *q*(ϑ|μ)] for the agent to find a sensory input $\tilde{\text{s}}$ caused by a certain ϑ. Friston and Stephan (2007) call this term expected Gibbs energy in virtue of its physical analogous quantity. The second term corresponds to the entropy of the variable causes ϑ. Therefore, conceptually, the latter formula is equivalent to the following relationship: *F* = *Expected energy* – *Entropy*, which has a bold resemblance to the Helmholtz and Gibbs free energy formulations in physics.

Friston's model borrows concepts and ideas from many different disciplines such as statistical physics, biology, microeconomics or machine learning among others (Friston, 2010). However, its equations can clearly be interpreted in information-theoretical terms. In fact, free energy is a concept from information theory used as a measure of evidence for a model. The infomax principle in statistics is just a particular case of the free energy minimization principle. This approach takes the same free energy concept but considering that the data are sensory inputs and the model is encoded by the brain. Furthermore, under simplifying assumptions, free energy can be regarded as just prediction error.

Remarkably, Friston's hypothesis has been already supported by empirical evidence. For example, it has been shown with EEG recordings that the free energy minimization principle was able to predict suppression of neural responses after repeated exposition (Moran et al., 2013). Besides, it is worth noting that there are a number of alternative computational models of cognition, for example the well-known ACT-R (Anderson, 1996). However, Friston's model provides a suitable description of learning processes in terms of information, which makes it more adequate for the framework proposed here. In addition, its equations show a bold resemblance to its thermodynamic analogous concepts, which allows to establish bridges with physically-principled models.

### Measuring the information content of brain activity and cognition

At this point, it is natural to ask from where information measures can be obtained in brain activity and cognition and whether this information is inherent to the stimuli or is rather generated in the brain computations. In fact, different choices of information measures may be suitable for distinct situations, objects of study or availability of the measures. For example, in primary cognitive processes such as visual perception the information content of the stimuli may be easily accessible. However, this approach is evidently not possible for studying higher cognitive processes like problem solving. Below we have exposed some approaches that have been applied so far and we have introduced some new ideas.

#### Method 1

Information measures can be directly obtained from the stimuli features. This method is especially meant for perceptual tasks where the relevant stimuli features for the task are easily accessible. Remarkably, Laughlin et al. (1998) applied this approach for the first time to obtain the energetic cost of processing one bit of information in blowfly visual sensory system. In particular, known amounts of information were delivered to the photoreceptors of blowfly retina in the form of fluctuations of light intensity. For a more detailed description of this method see Borst and Theunissen's (1999) review.

#### Method 2

**Figure 3:**
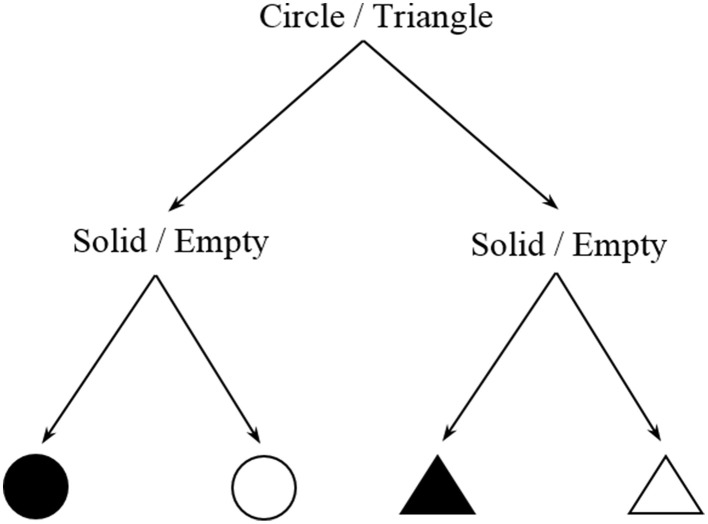
**Decision tree of a two-choice task**.

Information measures can be estimated directly from the complexity of the task. For a certain cognitive task there is often a finite number *N* of possible states of the system (intermediate steps, reactions, responses, etc…), all of them associated to a certain probability of occurrence. The basic idea is to compute Shannon entropy *H* = −Σ*p_i_log*_2_(*p_i_*) for a task. Intuitively, it is not equally costly to memorize the sequence ABBA [*I* = *log*_2_(2^4^) = 4 bits] as the sequence ABBBAABAABABAB [*I* = *log*_2_(2^14^) = 14 bits]. This method is analogous to the estimation of physical entropy with the Gibbs formula, as will be outlined in the following section. In this respect, there may be multiple combinations of intermediate steps (microstates) that lead to the same outcome or behavioral response (macrostate). In particular, the overall entropy or informational load of the task corresponds to the weighted average of the information content of all possible microstates i. Another simple hypothetical example is shown in Figure 3.

Two different choices, one about shape and another about color are represented. Assuming that the four outcomes are equiprobable, an information estimate of this task will be *log*_2_(*2*^2^) = *2* bits, or equivalently two yes/no questions to be answered in order to make a decision. Of course, this simplified description is just intended to give an intuitive idea of this method and does not pretend to accurately describe any nuances about color or shape that in real perceptual situations would drastically affect its information content. We would like to remark that this is a highly flexible method and exhaustive examination is required in order to depict a meaningful scheme that reasonably characterizes the task. For example, Stephen et al. (2009) employed a similar approach in an experiment where participants had to predict the rotation direction of the last one from a system of interconnected gears. However, in this case, random noise (i.e., unpredictability) was introduced in the task and taken as a measure of its information content.

#### Method 3

In the same way that neuroimaging techniques such as single cell recordings, fMRI and EEG can provide measures of energy consumption, these can also be used to infer the informational content of brain computations; that is, to estimate it directly from the neural code. In this case, the information content is inferred from the “complexity” of the data. Single cell recordings have been widely used for this purpose in animals (Borst and Theunissen, 1999) but it has recently been proposed that non-invasive methods such as fMRI or EEG can also be a suitable choice (Assecondi et al., 2015). In this respect, there are many different features that can be used to estimate the entropy from the raw data. Some examples are reaction times, the amplitude or latency of an EEG component, behavioral measures, parameters of the BOLD response in one or more voxels, etc. (Assecondi et al., 2015).

#### Method 4

Some algorithms in machine learning such as artificial neural networks (ANN) can be suitable to model learning processes in humans. As an advantage, the information cost of learning and the model parameters are easily accessible in these techniques. Interestingly, ANN do not only perform well in learning from data but also exhibit the actual neural biological structure of multiple interconnected neurons processing information. However, it cannot be inferred from this that the rest of the algorithm replicates the actual underlying biology. Indeed, the model is often fit by a (stochastic) gradient descent which is not intended to emulate any biological process. Nevertheless, it is reasonable to expect that ANN and the cost of actual brain computations will correlate if task difficulty changes. Modern versions of Perceptron (Rosenblatt, 1958), the backpropagation algorithm (Hecht-Nielsen, 1989) or Neocognitron (Fukushima, 1980) may be suitable choices to estimate the information cost of simple learning or perceptual tasks. Interestingly, a similar approach has already been successfully tested by Brunel et al. (2004) who used synaptic weights from a Perceptron model to estimate the amount of information processed by cerebellar Purkinje cells. For this, it was assumed that changes in synaptic efficacy underlie memory and learning, and their findings revealed that each Purkinje cell stores up to 5 kb of information. Another interesting choice may be EDLUT, a simulation software based on biologically plausible neural models very convenient to model experimental setups such as the implementation of a robotic arm (Ros et al., 2006). Finally, we deem appropriate the use of Friston's learning algorithms, which incorporate machine learning methods to a great extent and have proven accurate to fit neural data (Moran et al., 2013). In addition, this model has the advantage of having already been defined in information terms.

A crucial point in the first three approaches is that special attention must be paid in selecting the relevant features (of the task, the stimuli or the data) and their number of levels to be included in the entropy computations (Borst and Theunissen, 1999). The reliability of the information estimate will greatly depend on this choice. In other words, the definition of the number of symbols of our “alphabet” will have a considerable impact on the final Shannon entropy. In this respect, selecting a few informative features will often lead to a more accurate estimation than considering too many features without clear hypotheses about them (Assecondi et al., 2015).

## Connections between thermodynamics and information theory

### Classical connections between energy and information

As introduced above, Maxwell was the first to point out the possibility of exchanging information and physical entropy and since then many authors further studied this relationship (Feynman et al., 1998). Below, we have introduced some of the classical theoretical links between thermodynamics and information theory.

#### Gibbs and boltzmann formulas

Boltzmann (1877) provided the first definition of entropy from the statistical mechanics point of view. Boltzmann formulation assumes that there are *W* equiprobable microstates and the system is in thermodynamic equilibrium. In this situation, the entropy *S* of the system can be calculated as,

(7) S=kln(W)

where *k* is the Boltzmann constant, approximately 1.38 × 10^−23^ J/K. The number of microstates in physics refers to the number of all possible combinations of energetic states that the particles of the system can present. In general, this number will increase with the temperature (Feynman et al., 1965). Often, there will be a large number of microstates that lead to the same macrostate, i.e., to the same measurements of energy (E), volume (V), pressure (P), and temperature (T). It is important to note that Boltzmann's formula describes entropy in microscopic terms (microstates) and Classius's formula in macroscopic terms (heat exchange). Furthermore, Boltzmann formula nicely illustrates the third law of thermodynamics, namely that entropy is exactly zero at the temperature of 0 K. In this situation, there is a total absence of molecular vibration, leading to just one possible microstate.

More in general, Gibbs stated that the entropy of a system can be computed as follows (Feynman et al., 1998):

(8) $$S=-k\sum_{i}p_{i}\ln\left(p_{i}\right)$$

where *p_i_* corresponds to the probability of the microstate *i* taken from an equilibrium ensemble. We immediately notice two things. First, that Gibbs formulation has the same form as Shannon entropy except for a change of units given by the Boltzmann constant *k*. Second, in case all the microstates are equiprobable, and further assuming thermodynamic equilibrium, the Gibbs equation reduces into the Boltzmann formula. Intuitively, looking at Gibbs formula one can think of physical entropy as the number of yes/no questions that one needs to answer in order to completely specify the system's microstate, given that the system's macrostate is known. Importantly, Gibbs formula provides an informational reinterpretation of the second law of thermodynamics as follows: our information about an isolated system can never increase since spontaneous processes always entail a loss of information (Rothstein, 1951). Hence, according to Gibbs, a gain of entropy equals to a decrease of our information about the system.

#### Negentropy (brillouin)

As mentioned above, physical free energy is described by Helmholtz formula (Equation 1). Critically, Brillouin (1953) coined the term negentropy and linked this concept to information. He unified both, information and physical entropy under the same equation. Below, we have applied the same short reasoning as Brillouin's (1953) paper to deduce the formula. First, we consider a system with *P*_0_ different equiprobable structures (states). If we obtain information *I* about the system and we use it, as Maxwell's demon did with the gas, the number of possible structures is reduced to *P*_1_. Taking the natural logarithm of the ratio as a measure of information, this yields to *I* = $\tilde{K}$*ln(P*_0_/*P*_1_), where $\tilde{\text{K}}$ is a constant. For example, by choosing $\tilde{K}$ = log_2_(e) we would be measuring the information *I* = *log*_2_(*P*_0_/*P*_1_) in bits. By choosing $\tilde{K}$ = *k* (i.e., the Boltzmann constant), applying Boltzmann formula [*S* = *kln(P)*] and rearranging terms, the following relationship is obtained:

(9) $$S_{1}=S_{0}-I$$

where *I* corresponds to the negentropy term. Brillouin's idea of considering information and physical entropy as two interchangeable quantities has been widely accepted (Prigogine, 1978; Plenio and Vitelli, 2001; Maruyama et al., 2009). Remarkably, with Brillouin's equation, a generalization of the second law of thermodynamics can be formulated with the inclusion of an information term: (*S*_0_ − *I*) ≥ 0, for every isolated system (Brillouin, 1953). Furthermore, the latter statement entails a deeper result, namely that Shannon's source coding theorem and the second law are intrinsically related in the sense that a violation of one may be used to violate the other.

#### Processing information

Szilard (1929) proved that the act of acquiring information from a system generates entropy, or equivalently, it has an energetic cost due to the very nature of the procedure. He showed that the minimum amount of energy required to determine one bit of information is *kTln(2)* J or equivalently, an increase of entropy of *kln(2) J/K*. Later, Landauer (1961) studied the same phenomena in computation and found that the same minimum amount of heat is generated to erase one bit of information, e.g., in disk formatting. As an interesting fact, the generation of heat is the main reason that prevents modern computers from processing faster, and, in fact, these are still far from attaining Landauer's limit. Critically, the same minimum energetic costs of *kTln(2)* J per bit can be generalized to any other way of “manipulating” or processing information such as measuring, encoding, displaying, a yes/no decision, etc.

Interestingly, the former principles provide a solution for Maxwell demon's paradox, which is basically noticing that the demon cannot perform all the computations and measurements at zero cost. Thus, the demon must pay an energetic cost either for measuring (Szilard, 1929), for erasing (Landauer, 1961) or for encoding (Bennett, 1982) information in his memory. Alternatively, Maxwell's demon can be interpreted simply as a device changing negentropy into information and vice versa (Brillouin, 1953). Interesting conclusions from this paradox can also be drawn from the neuroscientific perspective, setting lower bounds for neural computations. In fact, the above considerations imply that it does not matter how the demon performs the computations in the brain, but these will have a minimum energetic cost of *kTln(2)* J per bit encoded or for every binary decision made (cold vs. hot molecule). The plausibility of Landauer's limit in the brain is discussed in the next section.

### Connections between energy and information in the brain

It is crucial to notice that the brain is not an exception to the previous constraints as it must obey the laws of thermodynamics. For example, De Castro (2013) analytically found the Landauer limit as the thermodynamic lower bound for brain computations. However, even though evolution is supposed to have “selected” the most energetically efficient processes, the physical lower bounds are not realistic quantities in the brain. Firstly, because the minimum processing unit considered in physics is the atom/molecule, which is distant from the actual way that brain operates; and, secondly, because neural networks incorporate important redundancy and noise factors that greatly reduce their efficiency (Narayanan et al., 2005).

Laughlin et al. (1998) was the first to provide explicit quantities for the energetic cost of processing sensory information. Their findings in blowflies revealed that for visual sensory data, the cost of one bit of information is around 5 × 10^−14^ Joules, or equivalently 10^4^ ATP molecules. Thus, neural processing efficiency is still far from Landauer's limit of *kTln(2)* J, but as a curious fact, it is still much more efficient than modern computers. We want to highlight that the previous quantities correspond to visual sensory processing in blowflies and thus these will greatly vary depending on the brain region, type of neuron or cognitive process (Howarth et al., 2010). Furthermore, neural noise and redundancy are topographic and population-size dependent and, in general, these quantities will increase with the size of the neural population (Narayanan et al., 2005).

Perhaps an even more challenging goal is estimating the neural information content when the data do not come from outside, as in sensory processing, but rather from internal representations (Collell and Fauquet, 2014). Deliberate thinking, problem-solving or creative thinking are instances of such tasks. In our opinion, it is especially convenient to approach these situations from the framework of “energy-information exchange” since, in this case, the information content can only be indirectly inferred. For example, by using some of the methods proposed in the previous section such as machine learning, simulation software or by estimating Shannon entropy of the task. Conversely, energetic measures can first be taken during the task execution (with EEG or fMRI) and use them later to infer its information content. For this conversion from energy into information, previous knowledge of the redundancy and noise factors of the involved brain regions must be taken into account (Collell and Fauquet, 2014). These inferred informational measures could be contrasted now with the estimated information independently obtained from machine learning or simulation software performing the same task. We suggest that initial tests should be conducted in simple perception tasks to gradually implement the method into increasingly complex cognitive tasks in case the preliminary results verified that estimated quantities correlate with the actual empirical measures. In this sense, perception is less subject to heuristics than high-order cognitive tasks. For example, there are many different ways to perform a mental arithmetic computation, leading to important differences in their computational costs. Remarkably, recent studies provide promising empirical evidence in favor of the previous hypotheses. Anderson and Fincham (2014) showed that multi-voxel pattern recognition could be used to discover mental states during the performance of a task. They were able to identify the different stages of solving a mathematical problem (encoding, planning, solving and responding) as well as to predict the processing duration in each stage by considering the difficulty and novelty of the task.

Cortical networks exhibit different modes of activity such as oscillations, synchrony and neural avalanches (Beggs and Plenz, 2003). This last phenomena is especially interesting to our work as it presents a deep relationship with a widely studied concept in physics referred to as criticality. Criticality is present in some physical systems composed of many nonlinear units interacting locally. Events such as earthquakes, forest fires, sand piles, nuclear chain reactions, neural activation, etc. are embedded in the aforementioned type of systems. These events are started by one unit exceeding a critical value (threshold) for some parameter and transferring the same change to adjacent units. As a result, a cascade of activity is propagated through a large number of units within the system. The spatial and temporal distributions of these events-near the critical value-are described by a power law, which by definition, entails scale invariance. That is, the same dynamics can be observed at many different scales. Crucially, experimental evidence tend to confirm that cortical brain activity exhibit a similar critical behavior, yielding to neural avalanches the size of which follow a power law (Beggs and Plenz, 2003; Haimovici et al., 2013). In addition, the brain operates near criticality most of the time. In this case, the critical parameter corresponds to the so-called branching parameter which is defined as the average number of descendants from one ancestor. That is, the average number of neurons activated in the next time bin given a single neuron being active in the current time bin. At the critical value, corresponding to a branching parameter equal to 1, neural avalanches recurrently occur and the information transmission capacity is maximized. By contrast, a subcritical network yields to an attenuated signal and a supercritical network to an epilepsy-like activation (Beggs and Plenz, 2003; Haimovici et al., 2013). It is worth noting the possibility to establish a connection between neural avalanches and free energy dynamics in the brain modeled as the quest to consume free energy in least time (Varpula et al., 2013). In fact, both models offer a description of the same event, i.e., activation of certain neural populations, and thus both frameworks must show consistency. Furthermore, criticality has also been object of study from a cognitive point of view, in this case, employing information-theoretic terms. In this respect, the critical value does not refer to any thermodynamic quantity but to Shannon entropy from the input. Stephen et al. (2009) provided an initial confirmation of the hypothesis that the emergence of new cognitive structures follows the same universal principles of criticality. They showed that after a certain entropic threshold, subject's understanding of the task experienced a discontinuous change in which they promptly gained insight on the task functioning. Essentially, it remains to be studied how the critical values from different perspectives: thermodynamic (Varpula et al., 2013), neural-branching (Beggs and Plenz, 2003) and cognitive-information theoretic (Stephen et al., 2009); are linked to each other. In addition, the presence of criticality in the brain and in cognitive dynamics has a repercussion on how information is processed and transmitted and thus especial attention must be paid when variables that produce discontinuous changes in the system are included in the model. However, as noted above, empirical evidence seems to confirm that brain operates near criticality most of the time, where information transmission is maximized (Beggs and Plenz, 2003; Haimovici et al., 2013).

### Connecting brain and cognitive models

Critically, thermodynamic and information-based models share some functional assumptions: (i) the brain must avoid phase transitions (i.e., drastic changes in its structure and function) as well as substantial entropy increases—if a large increase of entropy occurs, it must be rapidly removed; (ii) the amount of free energy (physical or informational) is always minimized in the least possible time in the brain; and, (iii) the brain encodes a probabilistic relationship between internal states, sensory inputs, behavioral responses and adaptive value of the outcomes of these behaviors (Kirkaldy, 1965; La Cerra, 2003; Friston, 2010; Del Castillo and Vera-Cruz, 2011; Varpula et al., 2013; Collell and Fauquet, 2014).

The above considerations suggest that both types of models stir the system's behavior in the same direction when the individual is exposed to a given situation. In other words, physical free energy within the brain varies in a similar manner to Friston's informational free energy. For example, let us suppose that the agent is facing a novel situation such as a new environment. Thus, because of the novelty of the situation, Friston's surprise term −*ln p*($\tilde{\text{s}}$|*m*) will be large, leading to an increase of free energy. Similarly, there will be a large amount of physical free energy to be consumed within the brain (ATP), mainly brought by an increased blood flow. This free energy will be rapidly reduced by the activation of the neural paths that consume more free energy in the least time, thus Δ*F_H_* < *0*. In a similar fashion, Friston's free energy *F* will be also rapidly lowered by the system taking action or by encoding the new information in the brain and thus avoiding surprising encounters in the future. Hence, a minimization of free energy Δ*F* < *0* is obtained too. Furthermore, a codification task has been carried out according to both models. That is, in Friston's model the system has reduced future prediction error by accurately updating the causes of the input. Analogously, from the thermodynamic viewpoint, the recently activated neural paths will be more easily retrieved in the future.

From a psychosocial perspective, adaptive behavior can be understood as a mechanism to reduce both, physical and Friston's entropies at the individual level. For instance, the existence of laws, establishing social bonds and public education are ultimately oriented to guarantee that the individual's entropy does not increase to a dangerous threshold. For example, a non-educated person will probably be exposed to a number of physically dangerous situations that an educated one would avoid. In this sense, Friston's entropy will also be higher for the non-educated individual since a large number of possible states can be occupied for most of the time. In other words, the fact that this individual will be more frequently exposed to surprising situations will be translated into an increase of Friston's entropy, which equals to the long-term average of surprise.

There are differences and resemblances between Friston's free energy *F* and Brillouin's free energy *I*. First of all, both terms define measures of information about the system as they take a negative logarithm of a probability distribution. In this sense, Friston's free energy corresponds to “statistical surprise” −*lnp*($\tilde{\text{s}}$|*m*) (plus a positive term), that is, how unlikely (i.e., informative) it is for the brain to receive a stimuli $\tilde{\text{s}}$. Frequent events are therefore encoded using fewer bits, i.e., virtually no new information for the individual. By contrast, highly unlikely events entail larger amounts of information to be encoded in the neural network in order to reduce surprise in future encounters. Similarly, Brillouin's negentropy term *I* also refers to a quantity of information that will increase if the system obtains/processes more information about the environment or about itself. Furthermore, Brillouin's free energy is not only a measure of information but, critically, it corresponds to actual free energy in the physical sense. Thus, Brillouin's formula *S*_1_ = *S*_0_ − *I* provides a measure (lower bound) of how much physical entropy *S*_1_ − *S*_0_ can be reduced within the brain by processing I bits of information, for example, by incorporating I bits from the environment. Thus, this analysis suggests that Brillouin's and Friston's free energies have a similar meaning in the neural processing context. However, it is clear that Friston's formulations constitute a “refinement” of Brillouin's free energy especially meant to be applied in self-organizing biological systems -in particular to the brain- and incorporate *ad hoc* parameters such as internal states μ. Nevertheless, unlike Brillouin's free energy, which is both an energetic and informational quantity, Friston's free energy does not have any thermodynamic explicit interpretation in any known units (e.g., J or J/K) but just shows a resemblance with Helmholtz equation (Equation 1). In our opinion, it is an interesting question to further study the thermodynamic implications and equivalences of Friston's information measures such as free energy and entropy.

### Spontaneity in the context of neural and cognitive activity

In a previous work we suggested that spontaneity can be an interesting topic to study in neuroscience (Collell and Fauquet, 2014). That is, to find conditions to determine what neural processes will naturally occur without external help given that we have knowledge of some of the system's variables. Spontaneous neural activity has been already object of study in neuroscience, mainly aimed at finding empirical evidence for neural avalanches. However, to our knowledge, no reference to information or thermodynamic measures has been made so far. Spontaneous processes are already well characterized in physics by the second law of thermodynamics. In addition, Brillouin (1953) generalized this principle and allowed for a definition of physical spontaneity in information terms. Similarly, spontaneous processes in chemistry are well-characterized by a version of the second law, i.e., the so-called Gibbs free energy formula Δ*G* = Δ*H* − *T*Δ*S*, where here *H* is the enthalpy (i.e., internal energy plus a positive term). In this respect, spontaneity is only possible if a process entails a decrease of Gibbs free energy Δ*G* ≤ *0*. Critically, these computations are feasible in practical situations and thus can be applied in order to make actual predictions. However, neuroscience does not have an operational description of spontaneous processes in terms of computable equations. On this direction, Varpula et al.'s (2013) principle of free energy consumption in the least time provides a theoretically well-grounded description of spontaneity at the neural level. However, it is unfeasible to use such free energy measures in order to make predictions about the occurrence of any cognitive or neural process. In fact, this would require real-time free energy recordings from different neural networks to verify that the electric signal indeed flows into the predicted path. Importantly, Beggs and Plenz (2003) provided a description of spontaneous neural activation, occurring at the critical value of the branching parameter, i.e., when it equals to 1. This characterization offers a promising description of spontaneity with a body of empirical support in the literature. Nevertheless, its initial formulation required single cell recordings from each single neuron within the network. In information-theoretic terms, Friston's model implicitly describes spontaneous processes as those that entail a decrease of informational free energy *F*. However, in practice, it is again not feasible to compute all the quantities in Equation (5) or (6) since some of them, such as hidden mental states or causes, are inaccessible for us and for the agent. For these reasons, the ideal scenario should allow for a definition in terms of both, information and thermodynamic measures. In this respect, Brillouin's formula (Equation 9) provides a powerful tool to plug information and energetic quantities in the same equation and indistinctly work with them. We suggest that interesting theoretical and empirical results could be derived from properly adapting Brillouin's formula to the brain processing context.

## Discussion

So far, we have reviewed the state of the art concerning thermodynamic models of brain activity (Prigogine, 1978; La Cerra, 2003; Varpula et al., 2013) and presented Friston's information-based model. In addition, we discussed and proposed means to obtain information measures from brain activity and cognition. Then, we put together some of the most important classical connections between information theory and thermodynamics. Furthermore, we analyzed conceptual nuances between the thermodynamic models of brain activity and Friston's model and connected them through the classical links between information and energy. In particular, we discussed the theoretical relationships between the free energy, surprise and entropy concepts from Friston's model and the equally termed concepts from thermodynamics.

The relationship between information and energy has been extensively studied in physics, chemistry and biology (Brillouin, 1953; Prigogine, 1978; Feynman et al., 1998; Del Castillo and Vera-Cruz, 2011). However, this connection has not been successfully formalized in neuroscience (Del Castillo and Vera-Cruz, 2011; Collell and Fauquet, 2014). In this respect, the main contribution of the present work is tackling the problem of relating brain activity and cognition from the classical connections between information theory and thermodynamics. In our opinion, neuroscience would greatly benefit from a better understanding of the trade-off, boundaries and exchange between information and energy in the brain and cognition. Furthermore, we believe that a proper formalization of how information and energy are linked in the brain will be essential in order to address philosophical and scientific questions such as the hard problem of consciousness, the relationship between brain and mind or the existence of free will. In addition, as we suggested above, this approach can be convenient in order to formally define the notion of spontaneity in brain and cognitive activity. To conclude, we aim at motivating more scientists to further study the relationship between brain and cognition from the framework proposed here. That is, to specify new mathematical models to link the existing thermodynamic- and information-based models, as well as to design empirical tests.

### Conflict of interest statement

The authors declare that the research was conducted in the absence of any commercial or financial relationships that could be construed as a potential conflict of interest.
